# Malaria transmission potential of *Anopheles gambiae* s.l. in indoor residual spraying areas with clothianidin 50 WG in northern Benin

**DOI:** 10.1186/s41182-024-00582-8

**Published:** 2024-02-09

**Authors:** Esdras Mahoutin Odjo, Mathilde Tognidro, Renaud Govoetchan, Antoine Abel Missihoun, Gil Germain Padonou, Juvenal Minassou Ahouandjinou, Bruno Akinro, Zinsou Come Koukpo, Filémon T. Tokponnon, Armel Djenontin, Clement Agbangla, Martin C. Akogbeto

**Affiliations:** 1 Centre de Recherche Entomologique de Cotonou, Cotonou, Benin; 2 Faculté des Sciences et Techniques de l’Université d’Abomey-Calavi, Abomey-Calavi, Benin; 3 Université de Parakou, Parakou, Benin; 4 Ecole polytechnique d’Abomey Calavi, Université d’Abomey-Calavi, Abomey-Calavi, Benin; 5 Direction Générale de la Recherche Scientifique, Ministère de l’Enseignement Supérieur et de la Recherche Scientifique, Cotonou, Benin

**Keywords:** *P. falciparum* infection, *Kdr-L995F*, *Ace-1 G280S*, Malaria, IRS, Benin

## Abstract

The study objective was to assess the frequency of the *kdr-L995F* and *ace-1 G280S* genetic mutations in *Anopheles gambiae* s.l. mosquitoes and examine their ability to transmit *Plasmodium falciparum* in areas where indoor residual spraying (IRS) was implemented with Clothianidin 50 WG. The study was conducted in six communes in the Alibori and Donga departments of which four were IRS-treated and two were untreated and served as control. Post-IRS monthly samples of adult mosquitoes were collected in study communes using human landing catches (HLC). *An. gambiae* s.l. specimens were processed to detect *kdr-L995F* and *ace-1 G280S* mutations via PCR as well as *Plasmodium falciparum* infectivity through CSP ELISA. Our data revealed a high and similar allelic frequency for the *kdr-L995F* mutation in both treated and control communes (79% vs. 77%, *p* = 0.14) whilst allelic frequency of the *ace-1 G280S* mutation was lower across the study area (2–3%, *p* = 0.58). The sporozoite rate was 2.6% and 2.4% respectively in treated and untreated communes (*p* = 0.751). No association was found between *Plasmodium falciparum* infection in *Anopheles gambiae* s.l. vectors and carriage of *kdr-L995F* and *ace-1 G280S* mutations regardless of genotypes. The study findings underline the need for an integrated approach to malaria control, combining different control methods to effectively target transmission. Regular monitoring of insecticide resistance and genetic mutations is essential to guide control strategies.

## Background

Malaria is a vector-borne disease transmitted by mosquitoes of the *Anopheles* genus [1]. In 2021, 95% of malaria cases (234 million/247 million) and 96% of deaths (593,000/619,000) were reported in the WHO African Region. Approximately 80% of all malaria-related deaths in the region involve children under 5-year old. This rate due to malaria deaths in children under 5-year old has remained constant in the region since 2015 [2]. The causative agent is a protozoan of the *Plasmodium* genus transmitted to humans through the bite of female mosquitoes [3]. In Benin, *Plasmodium falciparum* alone was responsible for 2,876,368 malaria cases recorded in 2021 and 2956 deaths with an increase 620 deaths compared to 2020 (2336) [2, 4]. The primary vector of this parasite in West Africa [5] and Benin [6] is *Anopheles gambiae* s.l. In the northern departments of Benin, malaria remains a significant public health issue [6].

Indoor residual spraying (IRS) is commonly used in Benin to complement the use of pyrethroid-impregnated mosquito nets to reduce malaria transmission in high-transmission areas (PMI, 2021). These interventions have been effective in reducing malaria transmission [7, 8]. Unfortunately, the expansion of vector resistance to pyrethroids (used in agriculture [9, 10], for impregnation of mosquito nets [11, 12] and for other domestic uses), carbamates and organophosphates (used for indoor residual spraying in Benin from 2008 to 2019 [13, 14]), poses a serious threat to progress in malaria control.

In 2021, Benin’s national malaria control program (NMCP) has opted to select Sumishield 50 WG, a dispersible granule formulation of clothianidin for large-scale IRS campaigns in the Djougou–Copargo–Ouake and Kandi–Gogounou–Segbana health zones [15]. This decision stems from the insecticide’s ability to act differently from previously used insecticides, particularly on vectors carrying the *kdr*-*L995F* and *ace-1 G280S* mutations [16]. Clothianidin, as a neonicotinoid insecticide, acts as an agonist of nicotinic acetylcholine receptors, causing paralysis and insect death [17]. In Benin, clothianidin-based insecticides have a long residual efficacy of 8 to 10 months in large-scale in community trials on mud and cement walls [18]. Its introduction into public health aims to optimize IRS by reducing infectivity through the elimination of malaria vectors, particularly those with the *kdr L995F* and *ace-1 G280S* resistance mechanisms. The *kdr*-*L995F* mutation in the voltage-dependent sodium channel (*Vgsc*), a target of pyrethroids and DDT, alters the interaction with insecticide molecules, resulting in knock-down resistance [19, 20]. The presence of this mutation at position 1014 in house flies (*Musca domestica*) was first documented [21]. In *Anopheles gambiae* s.l. mosquitoes, knock-down resistance mutations are found at position 995 [21–23]. They result from a leucine to phenylalanine substitution (L995F) in West Africa [24, 25] and a leucine to serine substitution (*L995S*) in Central and East Africa [26]. The *ace-1 G280S* mutation in acetylcholinesterase (AChE) makes resistant individuals less sensitive to the inhibitory action of organophosphates and carbamates [27]. Acetylcholinesterase (*AChE*) is an enzyme that terminates synaptic transmission by catalyzing the hydrolysis of the neurotransmitter acetylcholine. It has been demonstrated that the most common *ace-1* gene mutation in *Anopheles gambiae* s.l. results in the replacement of glycine (GGC) with serine (AGC) at codon 280 (G280S) [28]. This mutation of the acetylcholinesterase enzyme is also found at codon 119 in a partial crystal structure of the electric ray Torpedo californica [27, 29, 30]. AChE in resistant individuals is less sensitive to the inhibitory action of organophosphates and carbamates compared to that in susceptible individuals [31, 32]. In Benin, the resistance allele 280S has been consistently found at a low proportion in field populations of *An. gambiae* and *An. coluzzii* [13, 14]. These mutations are crucial as they are associated with insecticide resistance, particularly in *Anopheles gambiae* s.l. The frequency of these mutations in mosquito populations has a direct impact on the effectiveness of IRS programs [26, 33].

The need to assess the ability of resistant genotypes to transmit *Plasmodium falciparum* in communes under IRS with clothianidin 50 WG is essential to understand whether the use of clothianidin maintains significant efficacy against malaria vectors, despite the mutations observed.

## Methods

**Fig. 1:**
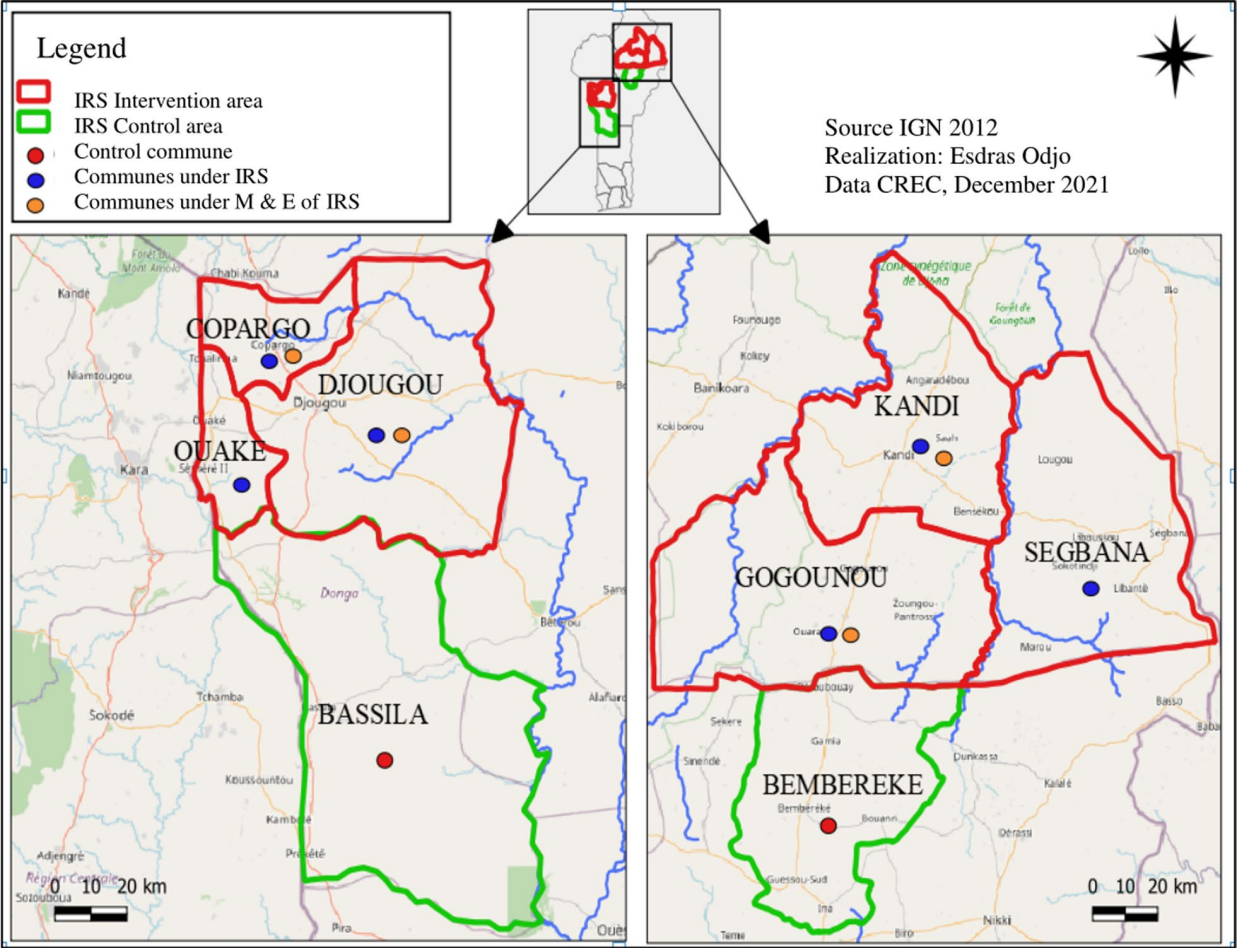
Map of the study area

The study was conducted in the health zones of Djougou–Copargo–Ouaké and Kandi–Gogounou–Segbana located respectively in Northwestern and Northeastern Benin where Clothianidin IRS has been implemented. Entomological monitoring and evaluation (M&E) data were collected in a total of 6 communes of which two treated communes and one neighbouring untreated commune to serve as control per department. Overall Copargo and Djougou in the Donga department and Kandi and Gogounou in Alibori were the treated communes surveyed whilst Bassila and Bembèrèkè were the control communes identified nearby (Fig. 1). Each commune is characterized by two seasons: a rainy season and a dry season. The population’s main activities are farming, livestock breeding and fishing [34–36].

### Sampling of adult mosquitoes

Post-IRS mosquito sampling was carried out on monthly basis between April and December in 2021 using the human landing catch (HLC). In both treated and control communes, mosquitoes were collected in two selected villages and four houses per village. Adult mosquitoes were captured of each visit by local volunteers. One collector inside and one collector outside were stationed at a household for hourly collections of mosquitoes at the level of each household.

Malaria vector mosquitoes collected were morphologically identified using taxonomic keys [37, 38]. The head and thorax were kept to process ELISA-CSP. The rest of the body (abdomen, wings, and legs) was used for genomic DNA extraction and molecular characterization.

### Screening of *P. falciparum* infection in *An. gambiae* s.l.

The heads-thoraxes of identified *An. gambiae* s.l. specimens were analysed using the CSP ELISA method following the protocol described by Wirtz et al*.* [39] to detect the circumsporozoite antigen (CSP), indicative of the presence of *Plasmodium falciparum*.

### DNA extraction of mosquito

Abdomens, wings, and legs were crushed in 200 μL of 2% cetyltrimethylammonium bromide (CTAB). After 5 min of water bath at 65 °C, 200 μL of chloroform was added to the mixture and centrifuged at 12,000 rpm for 5 min. The top portion was gently collected in another tube and supplemented with 200 μL of isopropanol, homogenized, and centrifuged again at 12,000 rpm for 15 min. The liquid in the tube was carefully inverted so as not to lose the pellet at the bottom. 200 µL of 70% ethanol were added to the pellet for precipitation. After 5 min of centrifugation, the contents of the tube were finely inverted again. The pellet was then drained for at least 3 h on the benchtop. The extracted DNA was reconstituted with 20 μL of sterile water and left in suspension on the benchtop overnight [40].

### Screening of *kdr-L995F* mutation in *An. gambiae* s.l.

Studies on insect species have revealed various substitutions of the *Vgsc* gene inducing a resistance phenotype [20, 41]. The PCR for detecting the kdr mutation employs four primers with distinct sequences: D1. ATAGATTCCCCGACCATG; D2. AGACAAGGATGATGAACC; D3. AATTTGCATTACTTACGACA; D4. CTGTAGTGATAGGAAATTTA [23].

The amplification program is composed of 40 cycles. Each cycle includes initial denaturation at 94 °C for 1 min, hybridization at 48 °C for 2 min, and elongation at 72 °C for 2 min. Finally, this PCR ends with a final elongation at 72 °C for 10 min [42].

### Screening of *ace-1 G280S *mutation in *An. gambiae* s.l.

The PCR for the *G280S* mutation utilizes two specific primers with the following base sequences: Ex3AGdir GATCGTGGACACCGTGTTCG and Ex3AGrev AGGATGGCCCGCTGGAACAG, according to the protocol by Weill et al. [41]. The amplification program was as follows: 30 cycles and each cycle included denaturation at 94 °C for 30 s, hybridization at 52 °C for 30 s, and elongation at 72 °C for 1 min. PCR products were digested with AluI restriction enzyme according to the manufacturer’s instructions before migration onto a 2% agarose gel.

### Clothianidin 50 WG

Insects possess nicotinic acetylcholine receptors (nAChR), which are the target of clothianidin in their nervous system [43]. These receptors are responsible for the transmission of nerve signals between nerve cells. Clothianidin acts by binding specifically to these nAChR receptors. When it binds to nAChR receptors, it activates them for an extended duration compared to acetylcholine, which is a natural neurotransmitter. This results in excessive stimulation of nerve cells and prolonged excitation of the insect’s nervous system [44]. Consequently, the insect becomes paralyzed because its nervous system remains constantly excited and can no longer function properly. Eventually, this leads to the insect’s death [43–45].

Clothianidin 50 WG was introduced for public health use in Benin in 2021 as part of large-scale community-based indoor residual spraying (IRS) campaigns. This choice aimed to optimize IRS by reducing infectivity through the elimination of malaria vectors, particularly those with resistance mechanisms such as *kdr-L995F* and *ace-1 G280S*, with the new chemical mode of action of clothianidin.

### Statistical analysis

Insecticide resistance mutations and *P. falciparum* infection data were analysed under R statistical software (version 4.1.0). The chi-square test for comparison of proportions was performed to verify the relationship between IRS-treated areas and untreated areas (control). The odds ratio was also evaluated using R software to assess the malaria transmission ability of different genotypes for *kdr-L995F* and *ace-1 G280S* mutations. Frequencies of the mutant allele *kdr L995F* and *ace-1 G280S* were calculated using the formula:

$$F\left(R\right)=\frac{2n{\text{RR}}+n{\text{RS}}}{2(n{\text{RR}}+n{\text{RS}}+n{\text{SS}})},$$

where *F*(*R*) is the frequency of resistance, *n* the number of mosquitoes of a given genotype, RR the homozygous resistant genotype, RS the heterozygous resistant genotype, and SS the susceptible genotype [14]. A multivariable logistic regression was done in RStudio version 1.3.959 and R statistical software version 4.21 to determine the association (odds ratio) between the independent parameters, (1) different genotypes for *kdr-L995F* and *ace-1 G280S* mutations (RR, RS, and SS), (2) location (Kandi, Gogounou, Bembèrèkè and Djougou, Copargo, Bassila).

## Results

Overall, 10,091 *An*. *gambiae* s.l. females, including 3585 and 6506 respectively from the two control communes and four IRS-treated communes, were processed for ELISA. However, a sub-sample of 2432 specimens of *An*. *gambiae* s.l. representing 24% of the total collected was analysed for insecticide resistance mutations.

### Frequency of *kdr-L995F* and *ace-1 G280S *mutations in *Anopheles gambiae* s.l.

Tables 1 and 2 present genotypic and allelic frequency variations of respectively *kdr-L995F* and *ace-1 G280S* mutations in mosquitoes of the *Anopheles gambiae* complex across the study communes. Pooled allelic frequency in IRS communes (79%) was higher than in control communes (77%, *p* = 0.14). At department level, *kdr-L995F* mutation showed generally high frequency in all the study communes, particularly in the communes of Donga department where a significantly lower frequency was found in the control commune Bassila (80%) compared to the two treated communes of Copargo (85%, *p* = 0.01) and Djougou (88%, *p* < 0.001) (Table 1). Meanwhile, the Alibori department showed significantly higher allelic frequency of *kdr-L995F* mutation in the control commune (Bembèrèkè) with similar frequency trend in Gogounou (72%, *p* = 0.67) unlike Kandi (68%, *p* = 0.03) (Table 1). Across all the study communes, homozygous resistant (RR) specimens for the *kdr-L995F* mutation were most prevalent, fluctuating between 70 and 83% in the Donga department and 54% to 59% in the Alibori department. Conversely, homozygous susceptible (SS) individuals were the least represented (Table 1).

**Table 1:** Frequency of the *kdr-L995F* mutation of the *Vgsc* gene in *An*. *gambiae* s.l. across the study communes

Department	Arm	Communes	Genotypic frequency % (*n*)			*F* (Kdr-L995F)	*p *value
			RR	RS	SS		
Donga	Control	Bassila	70 (309)	20 (89)	10 (43)	80	–
	IRS	Copargo	79 (242)	14 (42)	8 (24)	85	0.01
		Djougou	83 (459)	10 (56)	7 (36)	88	< 0.001
Alibori	Control	Bembèrèkè	59 (199)	29 (96)	12 (41)	74	–
	IRS	Gogounou	59 (254)	27 (116)	14 (61)	72	0.67
		Kandi	54 (196)	29 (106)	17 (63)	68	0.03
Total	Control		65 (508)	24 (185)	11 (84)	77	0.14
	IRS		70 (1151)	19 (320)	11 (184)	79	

*p *value: comparison of *An. gambiae* s.l. allelic frequencies (*F* (*R*)) between the treated and control communes, by department and for all departments (Test used: Chi-square test)

SS: susceptible homozygous; RS: heterozygous (resistant and susceptible hybrid); RR: resistant homozygous; %: percentage; (*n*): number of specimens analysed

**Table 2:** Frequency of the *G280S* mutation of the *ace-1* gene in *An*. *gambiae* s.l. across the study communes

Department	Arm	Communes	Genotypic frequency % (*n*)		*F* (G280S)	*p *value
			RS	SS		
Donga	Control	Bassila	5 (20)	95 (421)	2	–
	IRS	Copargo	6 (17)	94 (291)	3	0.66
		Djougou	5 (28)	95(523)	3	0.81
Alibori	Control	Bembèrèkè	5 (16)	95 (320)	2	–
	IRS	Gogounou	4 (19)	96 (412)	2	0.95
		Kandi	6 (23)	94 (342)	3	0.48
Total	Control		5 (36)	95 (741)	2	0.58
	IRS		5 (87)	95 (1568)	3	

*p *value: comparison of *An. gambiae* s.l. allelic frequencies (*F* (*R*)) between the treated and control communes, by department and for all departments (Test used: Chi-square test)

SS: susceptible homozygous; RS: heterozygous (resistant and susceptible hybrid); %: percentage; (*n*): number of specimens analysed

The allelic frequency of the *ace-1 G280S* mutation in *An*. *gambiae* complex mosquitoes was generally low (2–3%) in all communes and no significant difference was observed between control and IRS communes in both Alibori and Donga departments (Table 2). Susceptible homozygous specimens for the *ace-1 G280S* mutation were the most represented (94–96%). No resistant homozygous (RR) specimens were found in the study area (Table 2).

### Infectivity to *Plasmodium falciparum* in *An. gambiae* s.l.

**Fig. 2:**
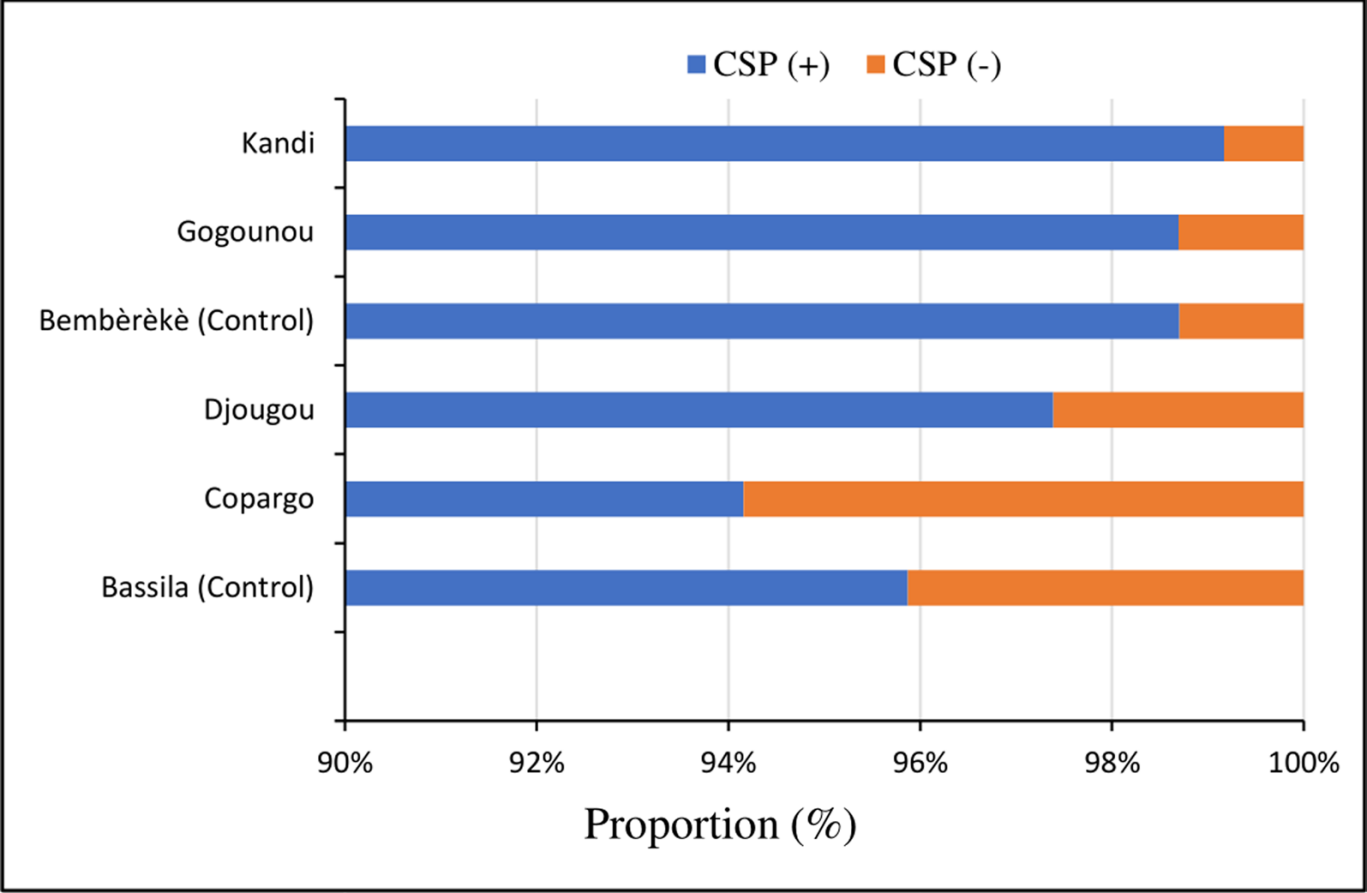
Sporozoite rate of *Anopheles gambiae* s.l. in IRS and control communes across the study area. CSP (+): *Anopheles gambiae* s.l. tested positive for the CS antigen of *Plasmodium falciparum*; CSP (−): *Anopheles gambiae* s.l. tested negative for the CS antigen of *Plasmodium falciparum*; s.l.: sensu lato

The assessment of infectivity to *Plasmodium falciparum* using the enzyme-linked immunosorbent assay (ELISA) targeting the circumsporozoite protein (CSP) was conducted on 10,091 *An*. *gambiae* s.l. Table 3 and Fig. 2 show variation of sporozoite rates in the study area. Pooled data showed no difference between untreated and treated communes across the study area (2.4% vs. 2.6%, *p* = 0.751). The trend was similar in the Alibori department with similar sporozoite rates in both untreated (1.3%) and treated communes (0.8–1.3%, *p* > 0.05) whilst in the Donga department, the treated Copargo commune provided a significantly higher sporozoite rate as compared to the control (5.8% vs. 4.1%, *p* = 0.049) unlike in Djougou where the sporozoite rate was lower than in the control (2.6% vs. 4,1%, *p* = 0.015).

**Table 3:** Sporozoite rate estimates of *Anopheles gambiae* s.l. in IRS and control communes

Department	Arm	Commune	*N* tested	*N* positive	SR (%)	*p *value
Donga	Control	Bassila	1429	59	4.1	–
	IRS	Copargo	1284	75	5.8	0.049
		Djougou	2181	57	2.6	0.015
Alibori	Control	Bembèrèkè	2156	28	1.3	–
	IRS	Gogounou	1839	24	1.3	1
		Kandi	1202	10	0.8	0.291
Total	Control		3585	87	2.4	0.751
	IRS		6506	166	2.6	

*N*: number of specimens; *N* positive: number of specimens positive for *P. falciparum*; %: percentage; SR: sporozoite rate

### Ability of *P. falciparum* transmission by *An. gambiae *s.l. in presence of *kdr-L995F* and *ace-1 G280S* mutations

Tables 4 and 5 present logistic regression analysis outcomes of the infectivity to *P*. *falciparum* in different genotypes for respectively the *kdr-L995F* and *ace-1 G280S* mutations across the study communes. Regarding the *kdr-L995F* mutation, the odds ratios (OR) showed similar *P*. *falciparum* infection in resistant homozygous (RR) and heterozygous (RS) specimens of *An*. *gambiae* s.l. in Donga regardless of the commune and study arm (OR: 1.26 with *p* = 0.52 in Bassila; OR: 1.37 with *p* = 0.41 in Copargo; OR: 1.9 with *p* = 0.13 in Djougou) (Table 4). In contrast, homozygous susceptible (SS) *An*. *gambiae* s.l. mosquitoes from Donga exhibited significantly higher likelihood for *P*. *falciparum* transmission compared to specimens of homozygous resistant (RR) genotype in all study communes (OR: 3.49 with *p* < 0.001 in Bassila; OR: 21.53 with *p* < 0.0001 in Copargo; OR: 5.67 with *p* < 0.0001 in Djougou) (Table 4). The same observation was made for all IRS communes compared with control communes. From the *kdr-L995F* mutation data collected in the Alibori department, the probability of *P*. *falciparum* infection in *An*. *gambiae* s.l. was generally similar irrespectively of the genotypes (RR, RS, SS) (*p* > 0.05) in Bembèrèkè, Gogounou and Kandi (Table 4).

**Table 4:** Ability of *P*. *falciparum* transmission in different genotypes of the *kdr-L995F* mutation across study communes

Department	Arm	Commune	Genotype	*P. falciparum* infection		OR	95% CI (OR)	*p *value
				Negative	Positive			
Donga	Control	Bassila	RR	275	34	1	–	–
			RS	77	12	1.26	0.56–2.64	0.5191
			SS	30	13	3.49	1.51–7.71	0.0005
	IRS	Copargo	RR	197	45	1	–	–
			RS	32	10	1.37	0.55–3.11	0.4055
			SS	4	20	21.53	6.77–90.75	< 0.0001
		Djougou	RR	422	37	1	–	–
			RS	48	8	1.90	0.72–4.46	0.1312
			SS	24	12	5.67	2.38–12.95	0.00005
Alibori	Control	Bembèrèkè	RR	185	14	1	–	–
			RS	87	9	1.37	0.50–3.54	0.4929
			SS	36	5	1.83	0.48–5.82	0.3354
	IRS	Gogounou	RR	238	16	1	–	–
			RS	115	1	0.13	0.003–0.86	0.0284
			SS	54	7	1.92	0.63–5.24	0.1732
		Kandi	RR	190	6	1	–	–
			RS	102	4	1.24	0.25–5.37	0.7449
			SS	63	0	0.00	0–2.64	0.3408
Total		Control	RR	460	48	1	–	–
			RS	164	21	1.23	0.67–2.16	0.4744
			SS	66	18	2.61	1.34–4.90	0.0025
		IRS	RR	1047	104	1	–	–
			RS	297	23	0.78	0.46–1.26	0.3677
			SS	145	39	2.71	1.75–4.12	< 0.0001

SS: susceptible homozygous; RS: heterozygous (hybrid resistant and susceptible); RR: resistant homozygous; %: percentage; *P.*: *Plasmodium*; OR: odds ratio

**Table 5:** Ability of *P. falciparum* transmission in the different genotypes of the *ace-1 G280S* mutation across study communes

Department	Arm	Commune	Genotype	*P. falciparum* infection		OR	95% CI (OR)	*p *value
				Negative	Positive			
Donga	Control	Bassila	RS	20	0	1	–	–
			SS	362	59	Inf	0.77–Inf	0.0909
	IRS	Copargo	RS	17	0	1	–	–
			SS	216	75	Inf	1.38–Inf	0.0159
		Djougou	RS	15	13	1	–	–
			SS	479	44	0.12	0.05–0.30	< 0.0001
Alibori	Control	Bembèrèkè	RS	16	0	1	–	–
			SS	292	28	Inf	0.34–Inf	0.3794
	IRS	Gogounou	RS	17	2	1	–	–
			SS	390	22	0.48	0.10–4.55	0.2861
		Kandi	RS	22	1	1	–	–
			SS	333	9	0.59	0.07–27.23	0.4828
Total		Control	RS	36	0	1	–	–
			SS	654	87	Inf	1.20–Inf	0.0259
		IRS	RS	71	16	1	–	–
			SS	1418	150	0.47	0.26–0.89	0.0152

SS: susceptible homozygous; RS: heterozygous (hybrid resistant and susceptible); %: percentage; *P.*: *Plasmodium*; OR: odds ratio; Inf: infinite

Regarding the *ace-1 G280S* mutation, whilst the communes of Alibori showed similar *P*. *falciparum* infection in hybrid (RS) and homozygous susceptible (SS) genotypes (OR: 0.48 with *p* = 0.28 in Gogounou; OR: 0.59 with *p* = 0.48 in Kandi), it was observed a significantly higher probability of *P. falciparum* infection in homozygous susceptible (SS) and heterozygous (RS) genotypes in the communes of Donga (*p* = 0.02 in Copargo; OR: 0.12 with *p* < 0.001 in Djougou) (Table 5).

## Discussion

The present study aimed to investigate the ability of *Anopheles gambiae* s.l. to transmit *P*. *falciparum* in the presence of *kdr-L995F* and *ace-1 G280S* mutations in Northern Benin. This study was conducted in six communes of which four communes were subject to IRS intervention and two communes were untreated. Our data revealed a relatively high allelic frequency of the *kdr-L995F* mutation across all study communes. These findings indicate the level of resistance of malaria vector *Anopheles gambiae* s.l. to the pyrethroids and organochlorine dichlorodiphenyltrichloroethane (DDT) within the study area. This aligns with the recent studies which demonstrated high allelic frequencies of the *kdr-L995F* mutation in various localities in Benin [12, 46, 47] and across different malaria-endemic African countries [48–51]. The widespread pyrethroid resistance is a consequence of the heavy selection pressure of intensified deployment of insecticide-treated bed nets in recent years [52]. In addition, the observed resistance situation in the surveyed sites is also linked to the extensive pyrethroid use in agriculture for crop protection, as reported by Yadouleton et al*.* [53, 54]. The allelic frequency of the *kdr-L995F* mutation within the *Vgsc* gene was significantly higher in the IRS communes than in the Donga department, unlike in the Alibori communes. This could be explained by the heavy use of pyrethroids in agriculture in the commune of Bembèrèkè and by IRS intervention in Copargo and Djougou. Indeed, although neonicotinoids act primarily on the insect nervous system by targeting nicotinic acetylcholine receptors, there is evidence that exposure to clothianidin can be associated with genetic mutations and metabolic resistance alleles in *An. gambiae* [55, 56].

Regarding the frequency of the *G280S* mutation within the *ace*-*1* gene, it remained very low across all communes, not exceeding 4%. However, it was observed an allelic frequency slightly lower in Bassila (control commune) than in Copargo and Djougou (IRS communes). Overall, the allelic frequencies of IRS communes (3%) and control communes (2%) were similar (*p* = 0.58), confirming the lesser targeting of the ace-1 gene in vector control interventions in Benin.

The infectivity of *An*. *gambiae* s.l. to *Plasmodium falciparum* mosquitoes significantly varied across communes and departments, regardless of the evolving frequencies of the *kdr-L995F Vgsc* and *G280S ace-1* genes mutation. Copargo commune showed significantly higher sporozoite rates (*p* = 0.049) compared to the other communes (Bassila and Djougou). The potential for increased malaria transmission observed in Copargo (treated zone) compared to the control commune (Bassila) could be attributed to the inherently higher malaria transmission in this area, as observed by Yadouleton et al*.* [57]. In fact, Copargo is a bordering commune of the Atacora department, where malaria incidence is amongst the highest nationally [6, 58].

The ability of *P*. *falciparum* transmission in *An*. *gambiae* s.l. carrying the *kdr-L995F* and *ace-1 G280S* mutations was investigated across all study communes. Our data found no association between *P*. *falciparum* infection and *kdr-L995F* mutation carriage in *An*. *gambiae* s.l. Thus, whether the mosquito is homozygous (RR), homozygous susceptible (SS), or heterozygous (RS), it does not impact its ability transmit *P*. *falciparum*. A similar trend was observed with the *G280S* mutation in the *ace-1* gene in the presence of which a comparable *P*. *falciparum* transmission was recorded irrespective of mosquito genotype. These findings are consistent with Ossè et al*.* [59] study demonstrating no significant difference in allelic frequency between *P*. *falciparum*-infected and non-infected *An*. *gambiae* s.l. female mosquitoes with all genotypes for the *kdr-L995F Vgsc* gene and *G280S ace-1* gene mutations. Similarly, Mitri et al. [60] reported linkage of genetic variation influencing *Plasmodium* infection to a natural 3-megabase haplotype on chromosome 2L carrying the *kdr* allele of the gene, which does not directly influence susceptibility to the parasite.

## Conclusion

The results of the investigations revealed that the *kdr-L995F* gene mutation is very frequent in the study area whilst the ace-1 mutation was less predominant in *An*. *gambiae* s.l. malaria vector within Donga and Alibori departments. Although malaria transmission was detected at variable levels across IRS and control communes, the results showed that the *ace-1 G280S* and *kdr-L995F* genes mutations have no impact on the transmission of *P. falciparum* by *Anopheles gambiae* complex members. This finding underscores the importance of adopting an integrated approach to malaria control, combining various control strategies to effectively target the vectors.

## Data Availability

The data used and/or analysed in this study are available from the corresponding author on reasonable request.

## Abbreviations

IRS: Indoor residual spraying
WHO: World Health Organization
SR: Sporozoite rate
HLC: Human landing catch
NMCP: National malaria control program
